# Contemporary applications of Y90 for the treatment of hepatocellular carcinoma

**DOI:** 10.1097/HC9.0000000000000288

**Published:** 2023-10-02

**Authors:** Qian Yu, Michael Khanjyan, Nicholas Fidelman, Anjana Pillai

**Affiliations:** 1 Department of Radiology, University of Chicago Medical Center, University of Chicago, Chicago, Illinois, USA; 2 Department of Radiology and Biomedical Imaging, University of California, San Francisco, California, USA; 3 Department of Medicine, Division of Gastroenterology, Hepatology, and Nutrition, University of Chicago Medical Center, University of Chicago, Chicago, Illinois, USA

## Abstract

Transarterial radioembolization (TARE) with yttrium-90 (^90^Y) microspheres has been widely adopted for the treatment of HCC. Recent advances in yttrium-90 (^90^Y) dosimetry have led to durable local responses. Radiation segmentectomy has become a viable alternative to thermal ablation for early-stage HCC (Barcelona Clinic Liver Cancer 0 and A) and has been commonly used as a bridge to transplant. TARE is also commonly used for downstaging to transplant using traditional lobar dosimetry and radiation segmentectomy techniques. Radiation lobectomy has a dual role in local tumor control and induction of contralateral liver lobe hypertrophy as a bridge to resection for patients with an inadequate future liver remnant. TARE continues to provide disease control for patients with limited vascular invasion and may be an alternative to systemic therapy for patients with localized advanced disease. The potential synergy between TARE and immunotherapy has been recognized, and prospective studies evaluating this combination are needed for patients with Barcelona Clinic Liver Cancer B and C HCC.

## Graphical abstract

**Figure:**
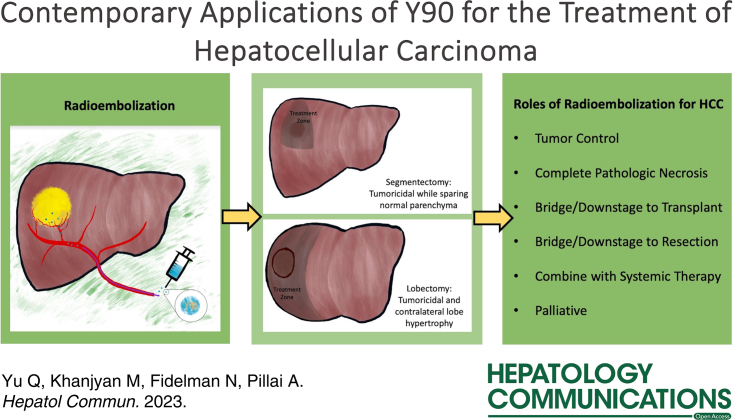

## INTRODUCTION

HCC is the most common primary hepatic malignancy worldwide and the leading cause of death among patients with cirrhosis.^1^ Although advancements in the treatment of HCC have prolonged survival, its incidence has continued to increase over the past 4 decades and poses a significant challenge to health care systems.^2^ Treatment of HCC can be divided into broad categories including surgical resection, liver transplantation, systemic therapy, and locoregional techniques that encompass percutaneous ablation and transarterial therapies including transarterial embolization, chemoembolization, and radioembolization (TARE) with yttrium-90 (^90^Y) microspheres. This review will focus on the many roles that TARE serves in the treatment paradigm for HCC with respect to early-stage, intermediate-stage, and advanced-stage disease, dosimetry considerations, and its role in bridging and downstaging patients to liver transplantation.

## TECHNICAL ELEMENTS OF RADIOEMBOLIZATION

### Yttrium-90

Yttrium-90 is a pure beta-particle emitter with an average particle energy of 0.94 MeV and a physical half-life of 64.2 hours. The average depth of tissue penetration by beta particles is 2.5 mm (range: 1–10 mm). ^90^Y decays at the deposition site to zirconium-90. For the purpose of delivery, ^90^Y is either physically embedded in 20–30 µm glass (TheraSphere, Boston Scientific, Natick, MA) microspheres or ionically bound to 20–60 µm resin (SIR-sphere, Sirtex Medical, Woburn, MA) microspheres.^3^ ^90^Y glass microspheres are approved for use in the United States for the treatment of HCC, while resin microspheres are approved for the treatment of colorectal liver metastases. The ^90^Y microspheres are delivered via a microcatheter into hepatic arterial circulation. The activity of individual ^90^Y microspheres is ~2500 Bq for glass and 50 Bq for resin microparticles on the day of calibration by the manufacturer.

Glass ^90^Y microspheres are available in activities ranging from 3 to 20 GBq and are approved for delivery within 12 days of the calibration date. The number of glass microparticles ranges from 1.2 to 8 million per vial. Glass microspheres are allowed to decay to achieve the desired dose on the delivery date. ^90^Y does not elute from glass microparticles, and as a result, there is no radioactivity in the body fluids. Resin microspheres may be delivered 1–4 days prior to the day of calibration with vial activities of 4.6–10 GBq and approximately 40–80 million particles per vial. The desired activity is aliquotted on the day of treatment. Less than 2% of ^90^Y elutes from the microparticles resulting in a small amount of activity in the blood and urine.^4^

Large ranges of available ^90^Y activities and numbers of microspheres allow considerable flexibility in selecting a dose with an appropriate number of microparticles. These decisions are guided by dosimetry considerations (delivery of a tumoricidal radiation dose while minimizing the amount of radiation to healthy liver parenchyma), size and vascularity of the target liver region, and the flow rates in the target hepatic artery branch. For example, subsegmental delivery of ^90^Y microspheres to a 2 cm tumor via a hepatic artery branch with an estimated blood flow rate of 0.5 mL/s would require a dose with fewer microparticles but higher activity than treatment of a 10 cm hypervascular mass via a right hepatic artery with a blood flow rate of 3 mL/s.^5^

### Patient selection and HCC staging

**FIGURE 1:**
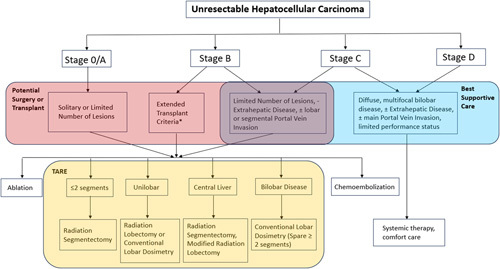
Application of transarterial radioembolization according to the Barcelona Clinic Liver Cancer staging. *Extended transplant criteria according to the criteria of the institution.^7^ Abbreviation: TARE, transarterial radioembolization.

The most widely accepted staging system for HCC is the Barcelona Clinic Liver Cancer (BCLC) system that includes 5 categories (0, A, B, C, and D) to classify patients ranging from early-stage HCC to advanced-stage HCC.^6^ The categorical divisions are based on the size and number of tumors, the patient’s performance status (derived from the Eastern Cooperative Oncology Group—ECOG score), and the Child-Pugh score that accounts for liver function. Stages 0 and A are classified as early, B as intermediate, and C and D as advanced stages. While, initially, TARE was reserved for the treatment of BCLC stage B and C HCC, it is increasingly being adopted for the treatment of BCLC stage A disease (Figure 1). Depending on disease burden and distribution, TARE may target liver regions ranging in size from <1 segment (subsegmental) to a single hepatic lobe or even the entire liver. Of note, whole liver TARE is generally not advised due to the increased risk of development of radioembolization-induced liver disease.^8^ Landmark ^90^Y studies are listed in Table 1, which will be further discussed in detail.

**TABLE 1:** Landmark clinical studies of TARE

Trial/year	Design	Sample size	Survival	Radiologic response	Major adverse event (%)	Key findings	Limitations
LEGACY 2021	Multicenter, retrospective, noncomparative	162	mOS: 57.9 mo 2-y OS: 94.8% 3-y OS: 84.6%	Objective response rate: 88.3% mTTP: not reached	19.1	TARE is safe and effective for treatment naive, solitary HCC measuring up to 8 cm.	Retrospective and noncomparative design. Relatively small sample size compared to systemic therapy trials.
RASER 2022	Prospective, single center, noncomparative	29	1-y OS: 96% 2-y OS: 96%	Objective response: 100% Complete response: 90% Median target tumor progression: not reached.	7	Radiation segmentectomy is safe and effective for unresectable very-early to early-stage HCC and is potentially curative.	Noncomparative study. Single institution. Small sample size. Strict inclusion limits generalizability.
TARGET 2021	Multicenter, retrospective, noncomparative	209	mOS: 20.3 mo	Objective response rate: 61.7%	20.6	Tumor-absorbed dose is associated with OS, objective response, and alpha-fetoprotein response.	Retrospective design. Heterogenous patient population, dosimetry calculation, and periprocedural medication across participating institutions. Limited availability of post-treatment dosimetry. Retrospectively determined multicompartment dosimetry. Lack of central review group for objective response evaluation.
TRACE 2022	Single-center, randomized controlled trial	72	TARE vs. DEB-TACE mOS (ITT): 30.2 vs. 15.6 mo	TARE vs. DEB-TACE mTTP (ITT): 17.1 vs. 9.5 mo mPFS:11.8 vs. 9.1 mo	TARE vs. DEB-TACE: 39 vs. 53%	TARE is associated with superior tumor control and survival than chemoembolization with comparable safety profile.	Slow participant accrual. Personalized dosimetry was not applied.
DOSISPHERE 2022	Randomized, multicenter, phase II trial	60	Personalized dosimetry vs. standard dosimetry: mOS (ITT): 26.6 vs. 10.7 mo. 1-y OS: 65.5% vs. 44.8% 2-y OS: 53.3% vs. 22.3%	Personalized dosimetry vs. standard dosimetry: mPFS (ITT): 6.0 vs. 3.4 mo 3-mo ORR (mITT): 71 vs. 36%.	Personalized dosimetry vs. standard dosimetry: 20% vs. 33%	Personalized dosimetry offers better radiologic response and survival than standard dosimetry and is associated with fewer adverse events.	Small sample size. Macroaggregated albumin as surrogate for microsphere is debatable. Only included tumors ≥7 cm. Questionable generalizability to resin microspheres.
SORAMIC 2019	Randomized, multicenter, phase II trial	424	TARE+sorafenib vs. sorafenib alone: mOS (ITT): 12.1 vs. 11.4 mo	Not reported	TARE+sorafenib vs. sorafenib alone: 64.8% vs. 53.8%	TARE+sorafenib did not offer survival benefit over sorafenib alone; potential effectiveness of adding TARE to sorafenib among noncirrhotic patients based on subgroup analysis.	Large proportion of patients did not receive allocated treatment or excluded from analysis due to major protocol deviation. Use of body surface area method for dose calculation and lack of 9mTc-MAA SPECT-CT for personalized dosimetry.
SIRveNIB 2018	Randomized, multicenter trial	360	TARE vs. sorafenib: mOS (ITT): 8.8 vs. 10.0 mo 1-y OS: 37.3 vs. 47.0%	TARE vs. sorafenib: mPFS: 5.8 vs. 5.1 mo; mTTP: 6.1 vs. 5.4 mo	TARE vs. sorafenib: 27.7% vs. 50.6%	TARE offers equivalent OS compared to sorafenib but is associated with lower adverse events.	More patients from the TARE group did not receive the assigned treatment. Heterogeneous patient population. Delayed treatment initiation in TARE group.
SARAH 2017	Multicenter, randomized, controlled, phase III trial	459	TARE vs. sorafenib: mOS (ITT): 8.0 vs. 9.9 mo 1-y OS: 39.5% vs. 42.1%	TARE vs. sorafenib: mPFS (ITT): 4.1 vs. 3.7 mo Disease control (best response): 68% vs. 78%	TARE vs. sorafenib: mOS: 77% vs. 82%.	For locally advanced or intermediate-stage HCC after unsuccessful TACE, TARE and sorafenib are comparable in OS and PFS, but TARE might better tolerated.	More patients from the TARE group did not receive the assigned treatment. Delayed treatment initiation in TARE group. No endpoint regarding tumor-/liver-absorbed dose.
CA 209-678 2021	Single-center, noncomparative, phase II trial	40	mOS: 16.9 mo 1-y OS: 33·0% 2-y OS: 14·1%	mPFS: 3.6 mo Objective response: 30.5% Disease control: 61.1%	6	TARE combined with nivolumab is safe	Heterogeneous patient population with various stages of disease and treatment history. Personalized dosimetry was not used in all patients. Noncomparative design. Single center.
SOLID 2023	Single-center, noncomparative, phase I/IIa	24	mTTP 15.2 mo.	mPFS 6.9 mo. Objective response: 83.3%.	8.7	TARE combined with durvalumab demonstrated safety and efficacy for locally advanced unresectable HCC.	Small sample size. Noncomparative, single-institution design.

Abbreviations: DEB-TACE, drug-eluting bead transarterial chemoembolization; ITT, intention-to-treat; mITT, modified intention-to-treat; mOS, median overall survival; mPFS, median progression-free survival; mTTP, median time to progression; ORR, objective response rate; OS, overall survival; PFS, progression-free survival; TACE, transarterial chemoembolization; TARE, transarterial radioembolization.

### Dosimetry

Radiation dosimetry for HCC evolved over time from the recommended delivery of ~120 Gy (range: 80–150 Gy) of absorbed tissue dose to a liver lobe. Ablative radiation doses targeting up to 2 liver segments may be administered through radiation segmentectomy (RS).^9^ Patients with large tumors limited to 1 liver lobe may benefit from radiation lobectomy, which aims to control tumor growth while promoting compensatory hypertrophy of the contralateral untreated liver lobe, possibly facilitating a future surgical resection.^9^

Personalized dosimetry is now routinely employed for the determination of the optimal tumor ^90^Y microsphere activity and the number of particles. Personalized dosimetry involves the calculation of the target liver volume and the use of mathematical models to estimate the absorbed tissue radiation dose.^5^ The most commonly employed method for the treatment of HCC is the Medical Internal Radiation Dose (MIRD) model.^10^ The MIRD dosimetry model assumes uniform radiation distribution within the target liver volume. This model is commonly used for both ^90^Y products. The MIRD model does not take into account the difference in microparticle distribution between tumor tissue and the surrounding liver parenchyma. The body surface area (BSA) method assumes a correlation between the BSA and liver size. It does not take into account target tumor volume or the difference in ^90^Y microparticle deposition in tumor and nontumor tissues. The BSA model has typically been used for treatment with ^90^Y resin microspheres and, however, is being employed less and less in favor of MIRD and partition models. Unlike MIRD and BSA models, the partition model takes into account both the target liver volume and differential microparticle distribution between tumor and nontumor compartments. Partition model dosimetry requires an accurate estimate of particle distribution ratio (tumor to normal, T:N ratio) between tumor and nontumor compartments, which is determined with the aid of commercially available software such as MIM (MIM Software, Beachwood, OH) and Simplicit90Y (Boston Scientific, Marlborough, MA). The T:N ratio may be estimated based on the Tc-99m MAA distribution in the target liver tissue or by evaluating tissue perfusion on intraprocedural cone beam CT. This testing is routinely performed during preparatory mapping hepatic angiography prior to TARE.

### RS for early-stage HCC

**FIGURE 2:**
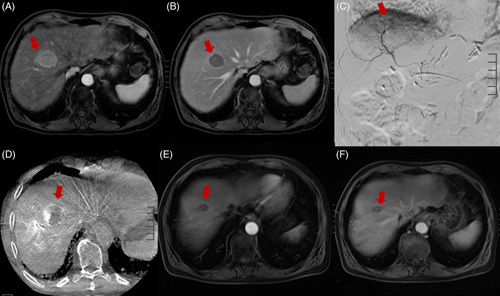
Imaging characteristics of early-stage HCC before, during, and after radiation segmentectomy. (A and B) Preradiation segmentectomy MRI images of a solitary, arterially enhancing hepatic segment 8 mass (A) demonstrating washout on portal venous phase (B) compatible with LI-RADS 5 HCC lesion (red arrow). (C and D) Angiographic digital subtraction image of segment 8 lesion at the time of radiation segmentectomy (C) with correlative, intraprocedural cone beam CT image (D) demonstrating lesion of interest (red arrow). (E and F) Approximately 6 months postradiation segmentectomy MRI images. Segment 8 lesion demonstrates no residual arterial enhancement with a slight decrease in size on portal venous phase (E) compatible with treated HCC (red arrow) (F). Abbreviation: LI-RADS, Liver Imaging Reporting and Data System

**FIGURE 3:**
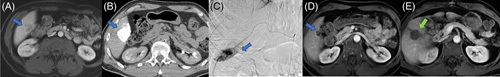
Patient with HBV-associated segment VI HCC (blue arrow) underwent radioembolization to bridge to surgery while undergoing staging work-up given high AFP > 1000. (A) MRI shows a 3.0 × 2.5 cm enhancing tumor. (B and C) Intraprocedural catheter-directed angiogram and CT demonstrated planned radioembolization coverage. (D) Postembolization MRI demonstrated a lack of enhancement and ablation cavity. (E) Surgical cavity after partial hepatectomy (green arrow). Abbreviation: AFP, Alpha-fetoprotein.

The gold standard for treatment of very-early-stage (BCLC 0) HCC and early-stage (BCLC-A) HCC is surgical resection. Ablation is an acceptable alternative for nonsurgical candidates. However, certain lesion locations within the liver and proximity to adjacent structures may not allow a safe percutaneous approach for ablation.^11^ Examples include tumors that are located centrally close to the liver hilum, in the caudate lobe, near the hepatic dome, adjacent to the heart, near the gallbladder or bowel, or adjacent to a major vessel. ^90^Y RS provides an endovascular alternative to ablation. RS involves the delivery of an ablative ^90^Y dose to up to 2 liver segments. Because of its safety and efficacy record, this technique has become an established alternative to ablation for the treatment of HCC.^12^ Examples of BCLC stage A HCC treated with RS are illustrated in Figures 2 and 3.

In 2011, Riaz et al^8^ published a study describing the safety and efficacy of RS for the treatment of unresectable HCC, which, at the time, was a novel technique. A total of 84 patients treated over a course of 5 years were included in the study, with 27 patients having BCLC-A stage disease. RS was defined as the treatment of 2 or fewer hepatic segments, and treatments were carried out with ^90^Y glass microspheres. The median dose delivered via a segmental hepatic artery was up to 521 Gy based on the MIRD model. Response to treatment was determined by contrast-enhanced CT or MRI at 1 month following TARE and every 2–3 months afterward utilizing the World Health Organization (WHO) criteria (partial response defined as ≥50% reduction in lesion size) and the EASL necrosis criteria (partial response defined as ≥50% reduction in tumor enhancement, hence viable tissue). The study demonstrated a WHO partial response in 57% and an EASL partial response in 81% of patients. The median time to progression (TTP) was 13.6 months, and median survival rates at 1, 2, and 3 years were 74%, 55%, and 27%, respectively. The authors recommended a minimum tumoricidal radiation dose of 205 Gy. Fatigue was the most common side effect (52% of patients). None of the patients in the study experienced radioembolization-induced liver disease. These favorable results laid the groundwork for future studies on RS as a viable approach to deliver high-dose radiation to tumors with minimal injury to normal hepatic tissue.^8^

RS was further evaluated by the RASER trial in 2022. The study enrolled 29 patients with very-early-stage or early-stage HCC deemed unfavorable for percutaneous ablation, who underwent RS with curative intent.^13^ Response was determined by imaging up to 24 months following treatment, utilizing the modified Response Evaluation Criteria in Solid Tumors criteria (partial response defined as at least 30% decrease in the sum of diameters of enhancing target lesions, taking as reference the baseline sum of the diameters of target lesions). The target dose was >205 Gy based on the MIRD model. Treatments were performed using ^90^Y glass microspheres. The trial demonstrated a complete tumor response in 83% and a partial response in 17% of patients. In patients with complete response, the median absorbed dose was 584 Gy (range: 181–3340 Gy). Survival 1 and 2 years following treatment were 96%. Target lesion progression was observed for 3 of 29 (10%) patients 7.6, 16.5, and 22.3 months following TARE. The cumulative incidence of any tumor progression was 14% at 1 year and 27% at 2 years. The most common side effect was fatigue, occurring in 31% of patients. The most common laboratory derangement was transient leukopenia, occurring in 45% of patients.^13^

Another landmark study in support of TARE for treatment for early-stage HCC was the multicenter retrospective LEGACY trial, which was published in 2021 and included 162 patients with solitary HCC less than 8 cm in size and median lesion size of 2.7 cm.^14^ ^90^Y glass microspheres were employed. Imaging response was assessed with modified Response Evaluation Criteria in Solid Tumors criteria. The median absorbed dose delivered was 410 Gy (IQR 200, 798 Gy). The objective response rate was 88.3% during 29.9 months of follow-up period, and 3-year overall survival (OS) was 86.6%. Common Terminology Criteria for Adverse Events grade 3 adverse events occurred in 19.1% of patients and primarily included self-limiting symptoms of fatigue and leukopenia.^14^

### Radiology-pathology correlation following RS at the time of transplantation

Pathologic evaluation at the time of liver transplantation following treatment with RS provides additional support for use of radioembolization in the treatment of HCC. In a multicenter study including 102 patients, Vouche et al^15^ studied the efficacy of RS with respect to radiology-pathology correlation in patients with solitary HCC <5 cm in size and not amenable to percutaneous ablation. Of the 102 treated patients, 33 were ultimately transplanted with a median time-to-transplant of 6.3 months, and liver explants were evaluated for necrosis. Complete pathologic necrosis (CPN) was defined as 100% tumor necrosis on hematoxylin and eosin staining, while partial necrosis was defined as 50%–99% necrosis. A total of 17 patients (52%) of patients demonstrated CPN, while the remaining 48% demonstrated >90% tumor necrosis. The study concluded that all patients treated with RS demonstrated 90%–100% pathology necrosis.^15^

Another multicenter study by Gabr et al^16^ published in 2021 evaluated 45 explants at the time of transplantation following RS for treatment of HCC <8 cm in size. Pathologic evaluation consisted of identifying viable neoplastic tissue, and response to treatment was categorized as CPN (no viable tumor), extensive necrosis (50%–99% necrosis), significant necrosis (minimal viable tissue), and partial necrosis (<50% necrosis). Of the 45 patients, 30 (67%) demonstrated CPN, 10 (22%) had extensive necrosis, and 5 (11%) showed partial necrosis. The degree of necrosis was correlated with absorbed radiation dose, whereas there was no correlation with tumor size. Twenty-four out of 28 patients (86%) who received a tumor-absorbed dose >190 Gy and all 11 patients receiving a dose >400 Gy demonstrated CPN. This study validated the findings by Vouche et al. and also established a target RS dose >400 Gy to reliably achieve complete tumor necrosis.^16^ These radiology-pathology correlation studies establish the efficacy of radioembolization for curative intent of HCC on par with percutaneous ablative techniques.

## INTERMEDIATE-STAGE AND ADVANCED-STAGE HCCs

The correlation between hepatic function and prognosis has been widely acknowledged, as observed in both prospective and retrospective studies.^17,18^ While selective delivery can be readily achieved in stage 0 and stage A HCC, radioembolization for stages B and C requires careful consideration of the patient’s performance status, disease burden, and future hepatic functional reserve. In these cases, higher tumor burden often leads to decreased survival, necessitating higher radiation doses and broader coverage. Striking the right balance between tumor response and preventing hepatic decompensation requires a combination of adequate tumor radiation dose and preservation of functional liver parenchyma. Thus, the concept of personalized dosimetry gained increasing popularity. In the DOSISPHERE-01 trial, a multi-institutional randomized phase II study of 60 patients with BCLC stage B (7 patients) and stage C (53 patients) HCC, the objective response rate was achieved in 71% and 36% of patients treated with personalized and standard dosimetry groups (*p* = 0.0074), respectively.^5^ According to the intention-to-treat analysis, the median OS rates were 26.6 and 10.7 months in the personalized (n = 31) and standard dosimetry (n = 29) groups (HR: 0.42, *p* = 0.0096), respectively. Patients with at least 1 tumor ≥7 cm (index lesion) were enrolled, with a mean index tumor size of 10.6 ± 2.8 cm and 11.1 ± 2.8 cm for personalized and standard dosimetry groups. The standard dosimetry group received 120 ±20 Gy to the perfused lobe, while the personalized dosimetry group received at least 205 Gy to the index lesion with a nontumor tissue dose below 120 Gy. In the personalized dosimetry cohort, grade 3 or greater adverse events were reported in 60% of patients compared to 76% of patients in the standard cohort.

These findings were validated by the multi-institutional retrospective TARGET study of 207 patients treated with TARE from 13 institutions around the world, which included 32.5% and 54.5% patients with BCLC stages B and C, respectively. Increased tumor-administered dose correlated with greater radiologic response (*p* = 0.044) and OS (median OS: 20.3 mo; OR per 100 Gy increase = 0.83, 95% CI: 0.71–0.95; *p* = 0.009).^19^

### TARE versus transarterial chemoembolization

Transarterial chemoembolization (TACE) is the established standard treatment for patients with intermediate-stage (BCLC B) unresectable HCC and preserved liver function. In comparison, TARE utilizes radiation as the primary tumoricidal modality while minimizing the embolic effect. Notably, in cases involving portal vein tumor thrombus (PVTT), TARE carries a lower risk of ischemic hepatic failure compared to arterial embolization in TACE. Consequently, portal vein occlusion is not a contraindication to TARE. Although several small prospective trials have explored the safety and effectiveness of TARE compared to TACE in the management of unresectable HCC,^20,21^ a comprehensive evaluation with larger sample sizes is still warranted.

One of the largest prospective studies comparing TARE with drug-eluting bead TACE is the TRACE study, which included 72 patients [61 (84.7%) patients with BCLC stage B and 11 BCLC stage A patients] who were not eligible for surgery or ablation from 2 institutions.^20^ TARE was performed using glass microspheres with a target absorbed dose of 120 Gy in the treated liver volume, preferably with selective delivery. In cases of bilobar disease, treatment was administered in 2 separate sessions, 30–45 days apart. TACE, on the other hand, was performed with 100–300 μm and 300–500 μm drug-eluting beads, with selective delivery and a maximum doxorubicin dose of 150 mg per session. The study revealed a similar safety profile between the 2 treatments. The median TTP was 17.1 months in the TARE arm compared to 9.5 months in the drug-eluting bead TACE arm (intention-to-treat analysis: HR = 0.36; 95% CI: 0.18, 0.70; *p* = 0.002; per-protocol analysis: HR = 0.29; 95% CI: 0.14, 0.60; *p* < 0.001). The median OS was 30.2 months for TARE and 15.6 months for drug-eluting bead TACE (HR = 0.48; 95% CI: 0.28, 0.82; *p* = 0.006). The incidence of grade 3 or worse adverse events was similar in both groups (39% and 53%, *p* = 0.47).

In an earlier single-center prospective randomized controlled trial of 45 patients with BCLC-A (35 patients, 78%) or BCLC B (10 patients, 22%), TTP (the primary outcome) was also longer in the TARE group than in the conventional TACE group (median TTP not reached > 26 mo vs. 6.8 mo; HR = 0.122; 95% CI: 0.027–0.557; *p* = 0.007).^21^ Moreover, the percentage of diarrhea and hypoalbuminemia was higher among TACE patients (21% vs. 0 and 58% vs. 4%). There was no statistically significant difference in terms of OS (18.6 vs. 17.7 mo, *p* = 0.99), which was not the primary outcome of the study.^21^

### Downstaging to resection

**FIGURE 4:**
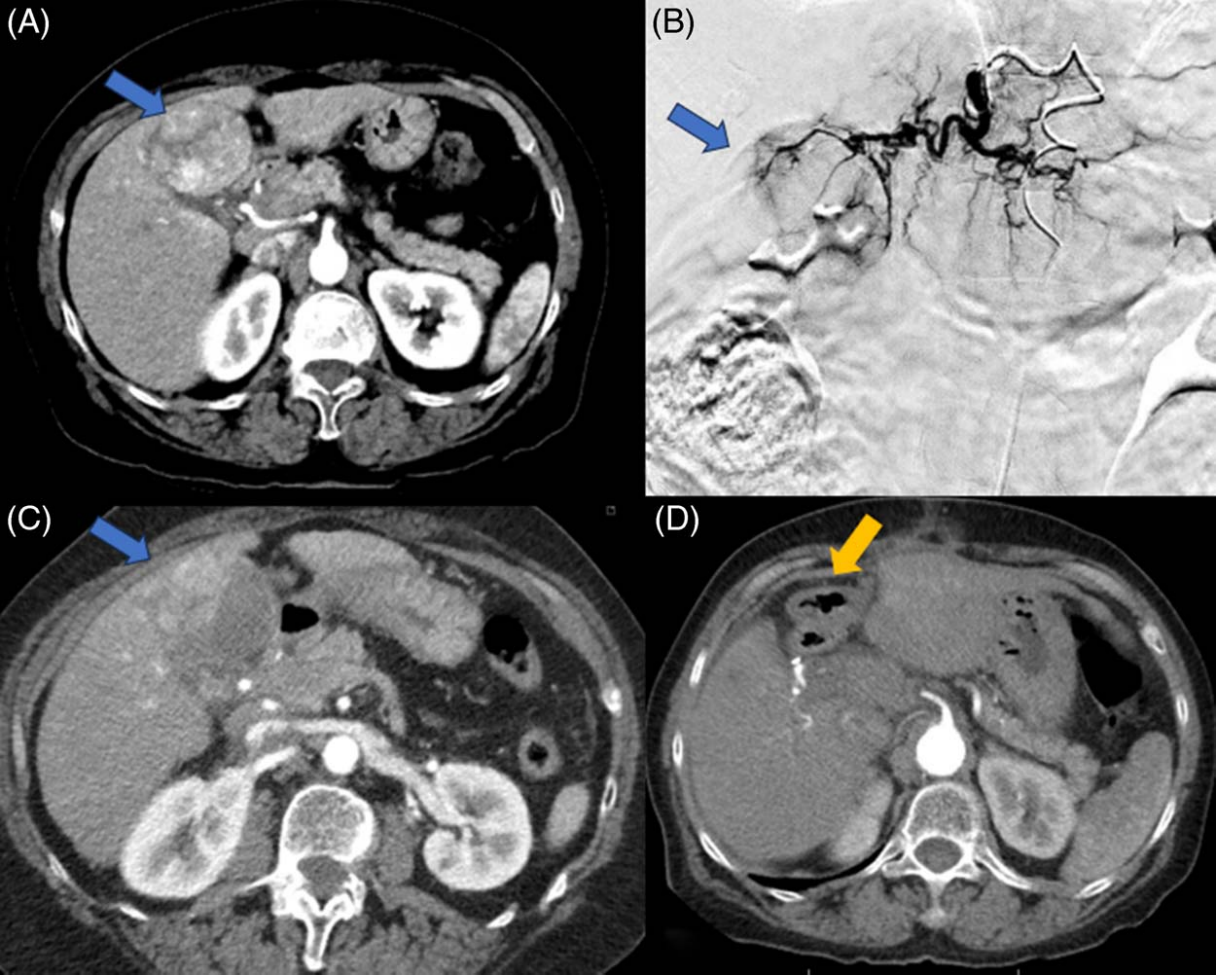
Patient with well-compensated hepatitis C cirrhosis and HCC situated in the central liver (blue arrow). (A) CT demonstrated arterial phase enhancing segment IVB tumor measuring 5.1 × 4.7 cm. (B) Catheter angiogram during selective radioembolization via segment IVB branch. (C) Postembolization CT shows partial response with the enhancing portion measuring 4.6 × 2.6 cm. (D) Patient underwent partial hepatectomy of segment IVB/V with a negative margin (yellow arrow: surgical bed).

TARE has demonstrated efficacy in downstaging initially unresectable HCC to a resection. For example, resection of larger tumors and those located in the central liver may be challenging due to involvement or close proximity of the middle hepatic vein.^22^ TARE effectively reduces tumor burden, enabling the conversion of initially unresectable HCC to resection (Figure 4). Lewandowski et al^23^ compared the efficacy of TACE and TARE for downstaging HCC from United Network for Organ Sharing (UNOS) T3 to T2, noting a higher partial response rate and a greater proportion of successful downstaging among patients that underwent TARE compared to TACE (58% vs. 31%). Similarly, Labgaa et al^24^ reported a 32% downsizing/downstaging rate among 349 HCC patients treated with TARE, with 22 patients undergoing liver transplantation and 10 patients undergoing resection. In another study of 118 patients treated with radioembolization, 6 out of 21 patients with UNOS T3 stage were downstaged, with 4 of them eventually receiving resection.^25^

The size of the future liver remnant (FLR) plays a critical role in determining patient eligibility for major hepatectomy. It is recommended to maintain an FLR of at least 40% for patients with chronic hepatitis or cirrhosis and an FLR of over 50% for those at a higher risk of liver decompensation.^22^ Radiation lobectomy employs ^90^Y microspheres (120–150 Gy using glass microspheres) to induce atrophy in the treated lobe while promoting simultaneous hypertrophy in the contralateral lobe, thereby enabling resection in patients initially considered ineligible due to insufficient FLR after resection.^26^ Although ^90^Y-induced FLR hypertrophy is typically slower than that achieved with portal vein embolization, it offers the advantage of potential downstaging and tumor control during the period between treatment and hypertrophy, often referred to as the “test-of-time.”^12,27,28^ Gabr et al^29^ observed a median FLR hypertrophy of 23.3% after TARE among 31 patients who eventually underwent partial hepatectomy. Similarly, Theysohn et al^30^ reported a 30.8% hypertrophy of the left hepatic lobe following right radiation lobectomy among 45 HCC patients with cirrhosis. Recently, a modified lobectomy technique combining a high segmental dose to the tumor and a lower lobar ^90^Y dose has been described as an approach to achieve tumor reduction and FLR hypertrophy simultaneously.^31,32^

### Downstaging to transplant

**FIGURE 5:**
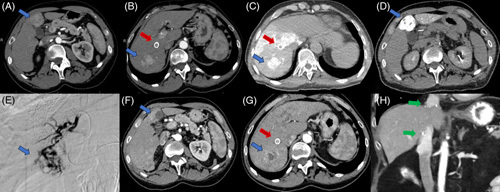
Patient with HCV cirrhosis and portal hypertension status post-TIPS (red arrow) presented with intermediate-stage HCC (blue arrows). (A) Segment IVB tumor measures 3.5 × 3.4 cm. (B) Segment VII tumor measures 3.9 × 3.0 cm. (C and D) Intraoperative hepatic arteriography–directed CT demonstrated planned radioembolization coverage with a target dose of 276 and 251 Gy, respectively. (E) Catheter angiogram selecting segment IVB tumor. (F and G) Postradioembolization arterial phase CT shows significantly decreased enhancement of target tumors. (H) Patient received liver transplant 4 months after radioembolization with follow-up CT 13 months post-transplant (green arrows indicate surgical anastomosis).

In cases where the disease burden is limited, the utilization of extended transplant criteria has demonstrated effectiveness in achieving post-transplant outcomes comparable to those under the Milan criteria. Various alternative criteria, including the University of California San Francisco (UCSF) criteria, Asan criteria, up-to-7 criteria, French alpha-fetoprotein model, and Metroticket 2.0 model, have shown similar survival rates and recurrence rates compared to the Milan criteria.^33–39^ Radioembolization has also proven effective in downstaging HCC and acting as a bridge to transplant. A retrospective study conducted at a single center, which involved 76 patients who were initially deemed ineligible for transplantation under the Milan criteria, revealed that 42% of patients became transplant eligible after undergoing TARE, while 58% remained noneligible.^40^ In a cohort of 207 patients who underwent liver transplant following TARE, 38 were successfully downstaged, and 169 were bridged to transplant, resulting in comparable OS and recurrence-free survival rates.^41^ An example of HCC downstaged to transplant is depicted in Figure 5.

### Portal vein thrombosis (PVT)

Patients diagnosed with HCC and cirrhosis accompanied by bland PVT face significant challenges in terms of treatment. Unless these patients exhibit preserved liver function, they are typically excluded from participating in clinical trials and receiving TACE. However, patients with HCC with PVT are not precluded from undergoing TARE, provided careful selection of suitable candidates and meticulous dosimetry planning. Somma et al^42^ reported their single-institutional prospectively collected data investigating the use of resin microspheres for TARE in patients with unresectable HCC, noting a comparable safety profile between patients with and without PVT given a lack of grade 3 or above adverse events in either group. Subgroup analysis of the SIRveNIB trial revealed lower OS among patients with PVT compared to those without PVT in the TARE groups (median OS intent to treat: 5.3 vs. 11.3 mo; per-protocol: 7.5 vs. 13.0 mo).^43^ In a phase 2 study involving 52 patients with intermediate and advanced HCCs, the median TTP and OS appeared lower in patients without PVT (median TTP 7 vs. 13 mo; median OS 13 vs. 18 mo) although statistical significance was not reached.^44^ Tumor response and Child-Pugh class were identified as independent factors associated with OS. Retrospective studies have emphasized the significance of baseline liver function, tumor burden, and patient performance status on OS,^45^ underscoring the importance of precise dosimetry and TARE planning to achieve selective and effective tumor-targeted radiation delivery while preserving healthy hepatic reserve.

### PVTT

The possibility of downstaging patients with PVTT to liver transplantation and resection through TARE exists but requires careful patient selection. A single-institutional study reported a successful downstaging rate of 5 out of 24 patients with PVTT to liver transplantation after ^90^Y resin TARE, with downstaged patients demonstrating higher tumor-absorbed radiation dose and lower pre-treatment AFP levels compared to those who did not undergo successful downstaging.^46^ In a propensity score-matched analysis involving 5 institutions and 65 HCC patients with intrahepatic PVTT, those treated with TARE (n = 41) exhibited significantly longer survival compared to those treated with sorafenib (n = 23; 20.3 vs. 9.1 mo, *p* = 0.001).^47^ Notably, 10 patients in the TARE group (24.4%) were successfully downstaged to liver transplant or hepatectomy. Similarly, Serenari et al^48^ showed the feasibility of TARE in downstaging HCC patients with PVTT to living donor liver transplant in 5 out of 17 patients (29.4%), leading to improved survival outcomes compared to patients without transplant (5-y survival: 60% vs. 0%, *p* = 0.03).

Selective ^90^Y administration and personalized dosimetry play a critical role in enhancing survival outcomes and maintaining a favorable safety profile in this patient population. In a retrospective study of 57 HCC patients with PVTT, those treated with ablative dose TARE demonstrated significantly improved OS compared to those treated with conventional dosimetry (median OS 45.3 vs. 18.2 mo, *p* = 0.003) while exhibiting similar hepatic toxicities.^49^

**FIGURE 6:**
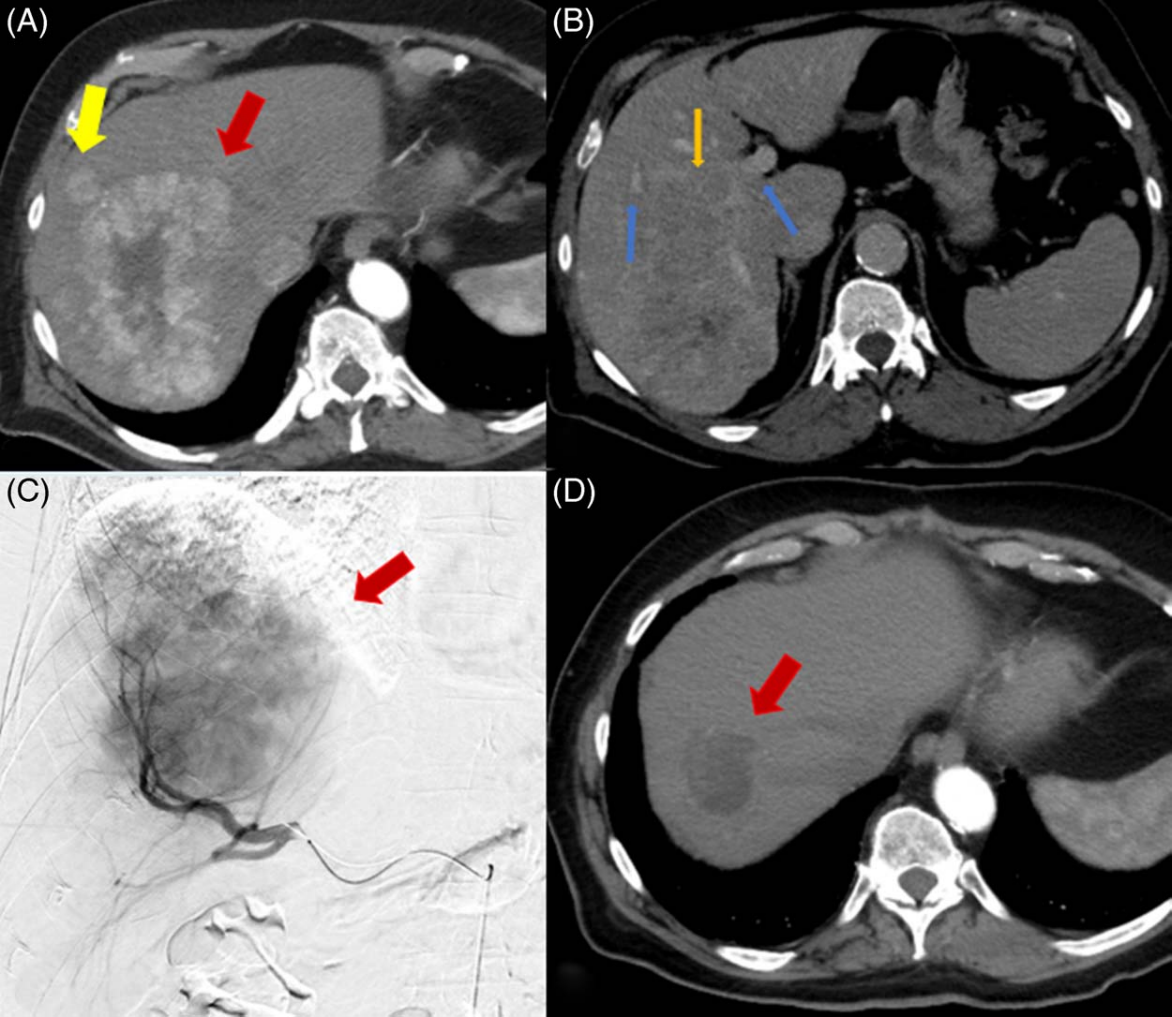
Patient presents with multifocal HCC. (A) Arterial phase CT shows a right hepatic lobe enhancing mass measuring 12.5 × 8.1 cm (red arrow) with an adjacent satellite lesion (yellow arrow). (B) Venous phase CT shows portal veins (blue arrow) and right portal vein invasion (orange arrow). (C) Right hepatic artery catheter-directed angiogram shows hypervascular right hepatic mass. (D) Arterial phase CT shows significantly decreased tumor size measuring 4.8 × 4.1 cm and decreased vascularity (red arrow) after right lobar radioembolization twice.

The extent of tumor vein invasion and baseline hepatic function are prognostic factors in this patient population. Bargellini et al^50^ observed an association between Milan PVTT score and OS, as patients with “good” and “dismal” scores demonstrated a median OS of 24.6 and 5.9 months, respectively. A meta-analysis primarily comprising retrospective studies evaluating TARE for HCC with PVTT indicated that median OS was worse in patients with Child-Pugh class B compared to class A (6.1 vs. 12.1 mo) and in patients with main PVTT compared to branch PVTT (6.1 vs. 13.4 mo).^51^ These findings suggested that patients with branch PVTT and adequate hepatic function might benefit from radioembolization. An example of HCC with PVTT treated with Y90 is shown in Figure 6.

### TARE versus systemic therapy

Systemic therapy is the standard treatment for BCLC stage B disease that is ineligible for extended liver transplant or TACE, as well as for BCLC stage C disease. TARE has demonstrated efficacy in patients who cannot tolerate systemic treatment or when used in combination with systemic therapy.

Three randomized controlled trials comparing TARE with sorafenib did not show the superiority of TARE. The SARAH (SorAfenib vs. Radioembolization in Advanced HCC) trial compared TARE and sorafenib but did not meet the primary endpoint of OS, despite a higher radiographic response rate in the TARE group (19% vs. 12%).^52^ The SIRveNIB trial (selective internal radiation therapy vs. sorafenib) also reported similar survival outcomes between the TARE and sorafenib groups, with a median OS of 8.8 and 10.0 months, respectively, but a more favorable safety profile in the TARE group (27.7% vs. 50.6% experiencing grade 3 or worse adverse events, *p* < 0.001).^43^ In the SORAMIC trial, the addition of TARE to sorafenib did not significantly improve OS compared to sorafenib alone in the intent-to-treat population.^53^ However, in the per-protocol population, a longer median OS was observed in the combination arm compared to the sorafenib arm although the difference was not statistically significant (14.0 vs. 11.1 mo, HR 0.86; *p* = 0.25). Grades 3–4 adverse events were more frequently reported in the selective internal radiation therapy + sorafenib group compared to the sorafenib alone. However, these trial results should be interpreted with caution due to the lack of data regarding tumor-absorbed doses and the absence of personalized dosimetry implementation.^54^ Post hoc analysis of the SARAH trial suggested a more favorable response in tumors treated with a higher radiation dose.^55^ Data from the DOSISPHERE and TARGET studies further confirmed the importance of personalized therapy in achieving treatment effectiveness while maintaining hepatic functional reserve.^5,19^ Future trials should incorporate such personalized TARE dosing regimens.

Moreover, there is a need for new clinical trials to evaluate the comparative or combined treatment of TARE with systemic regimens, as the landscape of systemic treatment has shifted from sorafenib to immunotherapy-based regimens as the first-line treatment for this disease stage. Imbrave150 trial showed that the combination of atezolizumab and bevacizumab (atezo/bev) is associated with improved median OS compared to sorafenib (19.2 vs. 13.4 mo).^56^ The HIMALAYA study demonstrated that the durvalumab and tremelimumab (durva/treme) regimen offers a better median OS of 16.4 months compared to sorafenib’s 13.8 months.^57^ Recently published phase 1/2a trial using combined TARE and durvalumab included 24 patients with BCLC stage B and C HCC without extrahepatic metastases, noting a median TTP of 15.2 months, median progression-free survival 6.9 months, and 18-month OS of 58.3% (95% CI: 36.4%–75.0%) with 2 (8.7%) grade 3 events of neutropenia and fever.^58^ The phase 2 single-center trial CA 209-678 evaluated a combination of ^90^Y and nivolumab among 40 patients with advanced HCC. The disease control rate was 61.1% with a median progression-free survival of 3.6 months and median OS of 16.9 months.^4,59^ Preliminary results from small case series have shown the potential benefits of combining atezo/bev and TARE for intermediate and advanced HCCs.^60^ Similarly, a prospective trial is underway to evaluate the effectiveness of adding the durva/treme regimen to TARE.^61^ Recently presented data from the MORPHEUS phase Ib/II randomized trial showed that the addition of TIGIT inhibitory immune-checkpoint tiragolumab to atezo/bev resulted in a higher objective response rate of 42.5% versus 11.1%, a longer progression-free survival of 11.1 months versus 4.2 months, with similar grade 3/4 adverse events (27.5% vs. 33.3%).^62^ The Imbrave050 study suggested that the adjuvant combination of atezo/bev after ablation or resection is associated with better recurrence-free survival compared to active surveillance alone [HR: 0.72 (95% CI: 0.56–0.93)].^63^ Further research is needed to determine if similar benefits can be replicated after TARE.

## CONCLUSIONS

TARE with yttrium-90 microspheres represents a promising and effective treatment modality for HCC at various stages. For early- and very-early-stage HCCs, RS is effective in achieving CPN and bridging to transplant or resection. For intermediate and advanced HCCs, personalized dosimetry has been increasingly utilized to achieve effective tumor radiation while preserving liver function, allowing downstaging to potentially curative resection or liver transplantation in selected cases. TARE is also efficacious for the treatment of select patients with advanced-stage HCC with vascular invasion. In the era of personalized dosimetry and immunotherapy, the integration of TARE and immune-checkpoint inhibitors appears promising and warrants further exploration through larger clinical trials (Figure 6).
